# A quick guide to survey research

**DOI:** 10.1308/003588413X13511609956372

**Published:** 2013-01

**Authors:** TL Jones, MAJ Baxter, V Khanduja

**Affiliations:** ^1^University of Cambridge,UK; ^2^Cambridge University Hospitals NHS Foundation Trust,UK

**Keywords:** Survey, Questionnaire, Design, Research, Guide

## Abstract

Questionnaires are a very useful survey tool that allow large populations to be assessed with relative ease. Despite a widespread perception that surveys are easy to conduct, in order to yield meaningful results, a survey needs extensive planning, time and effort. In this article, we aim to cover the main aspects of designing, implementing and analysing a survey as well as focusing on techniques that would improve response rates.

Medical research questionnaires or surveys are vital tools used to gather information on individual perspectives in a large cohort. Within the medical realm, there are three main types of survey: epidemiological surveys, surveys on attitudes to a health service or intervention and questionnaires assessing knowledge on a particular issue or topic.^1^

Despite a widespread perception that surveys are easy to conduct, in order to yield meaningful results, a survey needs extensive planning, time and effort. In this article, we aim to cover the main aspects of designing, implementing and analysing a survey as well as focusing on techniques that would improve response rates.

## Clear research goal

The first and most important step in designing a survey is to have a clear idea of what you are looking for. It will always be tempting to take a blanket approach and ask as many questions as possible in the hope of getting as much information as possible. This type of approach does not work as asking too many irrelevant or incoherent questions reduces the response rate^2^ and therefore reduces the power of the study. This is especially important when surveying physicians as they often have a lower response rate than the rest of the population.^3^ Instead, you must carefully consider the important data you will be using and work on a ‘need to know’ rather than a ‘would be nice to know’ model.^4^

After considering the question you are trying to answer, deciding whom you are going to ask is the next step. With small populations, attempting to survey them all is manageable but as your population gets bigger, a sample must be taken. The size of this sample is more important than you might expect. After lost questionnaires, non-responders and improper answers are taken into account, this sample must still be big enough to be representative of the entire population. If it is not big enough, the power of your statistics will drop and you may not get any meaningful answers at all. It is for this reason that getting a statistician involved in your study early on is absolutely crucial. Data should not be collected until you know what you are going to do with them.

## Directed questions

After settling on your research goal and beginning to design a questionnaire, the main considerations are the method of data collection, the survey instrument and the type of question you are going to ask. Methods of data collection include personal interviews, telephone, postal or electronic (Table 1).

Collected data are only useful if they convey information accurately and consistently about the topic in which you are interested. This is where a validated survey instrument comes in to the questionnaire design. Validated instruments are those that have been extensively tested and are correctly calibrated to their target. They can therefore be assumed to be accurate.^1^ It may be possible to modify a previously validated instrument but you should seek specialist advice as this is likely to reduce its power. Examples of validated models are the Beck Hopelessness Scale^5^ or the Addenbrooke’s Cognitive Examination.^6^

The next step is choosing the type of question you are going to ask. The questionnaire should be designed to answer the question you want answered. Each question should be clear, concise and without bias. Normalising statements should be included and the language level targeted towards those at the lowest educational level in your cohort.^1^ You should avoid open, double barrelled questions and those questions that include negative items and assign causality.^1^ The questions you use may elicit either an open (free text answer) or closed response. Open responses are more flexible but require more time and effort to analyse, whereas closed responses require more initial input in order to exhaust all possible options but are easier to analyse and present.

**Table 1:** Advantages and disadvantages of survey methods

Method of data collection	Advantages	Disadvantages
Personal	• Complex questions	• Expensive
	• Visual aids can be used	• Time inefficient
	• Higher response rates	• Training to avoid bias
Telephone	• Allows clarification	• No visual aids
	• Larger radius than personal	• Difficult to develop rapport
	• Less expensive or time consuming	
	• Higher response rates	
Postal	• Larger target	• Non-response
	• Visual aids (although limited)	• Time for data compilation
	• Lower response rates	
Electronic	• Larger target	• Non-response
	• Visual aids	• Not all subjects accessible
	• Quick response	
	• Quick data compilation	
	• Lower response rates	

## Questionnaire

Two more aspects come into questionnaire design: aesthetics and question order. While this is not relevant to telephone or personal questionnaires, in self-administered surveys the aesthetics of the questionnaire are crucial. Having spent a large amount of time fine-tuning your questions, presenting them in such a way as to maximise response rates is pivotal to obtaining good results. Visual elements to think of include smooth, simple and symmetrical shapes, soft colours and repetition of visual elements.^7^

Once you have attracted your subject’s attention and willingness with a well designed and attractive survey, the order in which you put your questions is critical. To do this you should focus on what you need to know; start by placing easier, important questions at the beginning, group common themes in the middle and keep questions on demographics to near the end. The questions should be arrayed in a logical order, questions on the same topic close together and with sensible sections if long enough to warrant them. Introductory and summary questions to mark the start and end of the survey are also helpful.

## Pilot study

Once a completed survey has been compiled, it needs to be tested. The ideal next step should highlight spelling errors, ambiguous questions and anything else that impairs completion of the questionnaire.^8^ A pilot study, in which you apply your work to a small sample of your target population in a controlled setting, may highlight areas in which work still needs to be done. Where possible, being present while the pilot is going on will allow a focus group-type atmosphere in which you can discuss aspects of the survey with those who are going to be filling it in. This step may seem non-essential but detecting previously unconsidered difficulties needs to happen as early as possible and it is important to use your participants’ time wisely as they are unlikely to give it again.

## Distribution and collection

While it should be considered quite early on, we will now discuss routes of survey administration and ways to maximise results. Questionnaires can be self-administered electronically or by post, or administered by a researcher by telephone or in person. The advantages and disadvantages of each method are summarised in Table 1. Telephone and personal surveys are very time and resource consuming whereas postal and electronic surveys suffer from low response rates and response bias. Your route should be chosen with care.

Methods for maximising response rates for self-administered surveys are listed in Table 2, taken from a Cochrane review.2 The differences between methods of maximising responses to postal or e-surveys are considerable but common elements include keeping the questionnaire short and logical as well as including incentives.

**Table 2:** Methods for improving response rates in postal and electronic questionnaires^2^

Postal	Electronic
Monetary or non-monetary incentives	Non-monetary incentives
Teaser on the envelope	Personalised questionnaires
Pre-notification	Include pictures
Follow-up with another copy included	Not including ‘survey’ in subject line
Handwritten addresses	Male signature
University sponsorship	White background
Use recorded delivery	Short questionnaire
Include return envelope	Offer of results
Avoid sensitive questions	Statement that others have responded

**Key points**

– Involve a statistician early on.
– Run a pilot study to uncover problems.
– Consider using a validated instrument.
– Only ask what you ‘need to know’.
– Consider guidelines on improving response rates.

## Analysis

The collected data will come in a number of forms depending on the method of collection. Data from telephone or personal interviews can be directly entered into a computer database whereas postal data can be entered at a later stage. Electronic questionnaires can allow responses to go directly into a computer database. Problems arise from errors in data entry and when questionnaires are returned with missing data fields. As mentioned earlier, it is essential to have a statistician involved from the beginning for help with data analysis. He or she will have helped to determine the sample size required to ensure your study has enough power. The statistician can also suggest tests of significance appropriate to your survey, such as Student’s t-test or the chi-square test.

## Conclusions

Survey research is a unique way of gathering information from a large cohort. Advantages of surveys include having a large population and therefore a greater statistical power, the ability to gather large amounts of information and having the availability of validated models. However, surveys are costly, there is sometimes discrepancy in recall accuracy and the validity of a survey depends on the response rate. Proper design is vital to enable analysis of results and pilot studies are critical to this process.
